# Live birth rate per transfer is not impacted by the proportion of smooth endoplasmatic reticulum aggregates oocytes

**DOI:** 10.52054/FVVO.15.2.070

**Published:** 2023-06-30

**Authors:** A Spileers, I De Croo, D Stoop, F Vanden Meerschaut

**Affiliations:** Ghent University Hospital, Centre for Reproductive Medicine, Corneel Heymanslaan 10, Ghent 9000, Belgium

**Keywords:** ICSI, live birth rate, smooth endoplasmatic reticulum aggregates, SERa

## Abstract

**Background:**

Despite the data published to date, prognostic factors and the clinical impact of ICSI cycles with smooth endoplasmatic reticulum aggregates (SERa) positive oocytes remain unclear.

**Objective:**

Are the clinical outcomes of an ICSI cycle impacted by the proportion of oocytes with SERa?

**Materials and Methods:**

Retrospective study (2016-2019), including data from 2468 ovum pick-ups, performed in a tertiary university hospital. Cases are categorised based on the rate of SERa positive oocytes compared to the total number of MII oocytes: 0% (n=2097), <30% (n=262) and ≥30% (n=109).

**Main outcome measures:**

Patient characteristics, cycle characteristics and clinical outcomes are compared between the groups.

**Results:**

Compared to SERa negative cycles, women with ≥30% SERa positive oocytes are older (36.2y vs. 34.5y, p<0.001), have lower anti-mullerian hormone levels (AMH) (1.6ng/ml vs. 2.3ng/ml, p<0.001), have received more gonadotropins (3227U vs. 2858IU, p=0.003), have a lower number of good quality day 5 blastocysts (1.2 vs. 2.3, p<0.001) and face more blastocyst transfer cancellation (47.7 vs. 23.7%, p<0.001). Women with <30% SERa positive oocytes are younger (33.8y, p=0.04), have higher AMH levels (2.6ng/ml, p<0.001), have more oocytes retrieved (15.1, p<0.001), have a higher number of good quality day 5 blastocysts (3.2, p<0.001) and have less transfer cancellations (14.9%, p<0.001) compared to SERa negative cycles A multivariate analysis shows no significant difference in cycle outcomes between the categories.

**What is new?:**

Treatment cycles with ≥30% SERa positive oocytes are less likely to result in an embryo transfer when only non-SER oocytes are used. However, live birth rate per transfer is not affected by the proportion of SERa positive oocytes.

## Introduction

**Figure 1:**
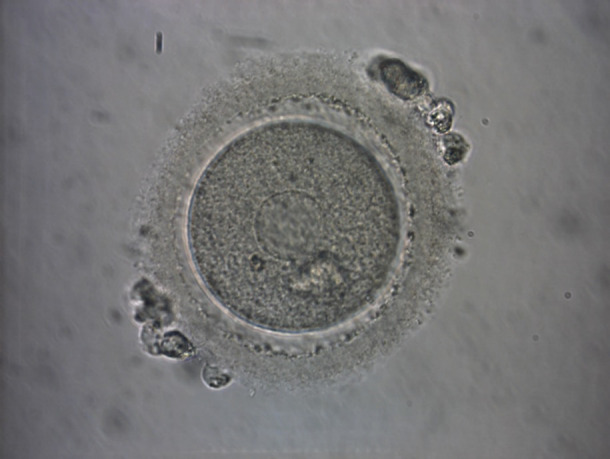
Oocyte with smooth endoplasmatic reticulum aggregates (arrow).

A smooth endoplasmic reticulum aggregate (SERa) is an intracytoplasmic dysmorphism in metaphase II (MII) oocytes in which the aggregations appear as round flat discs in the ooplasm (Figure 1).

The presence of SERa disrupts the normal functioning of smooth endoplasmic reticulum. The calcium signalling appears to be impaired in SERa positive oocytes, which plays an important role in oocyte maturation, fertilisation, and embryonic development (Ferreux et al., 2019; Mateizel et al., 2013; Alpha Scientists in Reproductive Medicine, 2011). Dal Canto et al. (2017) and Otsuki et al. (2018) identified an increase in meiotic and mitotic division problems that may eventually lead to aberrant embryo division due to the spindle size and cortical actin structure that may be disrupted in the SERa positive oocytes.

Several case series published abnormal fetal and neonatal outcomes after transfer from a SERa positive cycle. The reported congenital malformations are very diverse. There are cases with a complex chromosomal rearrangement with 2q31 deletion (Sfontouris et al., 2018), Beckwith Wiedemann syndrome (Otsuki et al., 2004), ventricular septal defect (Sá et al., 2011), holoprosencephaly, multiple malformations (Akarsu et al., 2009), hernia diaphragmatic (Ebner et al., 2008), trisomy 21, brachialis paresis, hypospadias, trisomy 18 and double kidney (Mateizel et al., 2013). Some of these congenital abnormalities were described when a non-affected embryo (originating from a non-affected oocyte) originating form a SERa positive cycle was transferred.

Given the theoretical risk of congenital disease, the Istanbul Consensus recommended in 2011 not to inject and therefore no longer use SERa positive oocytes (Alpha Scientists in Reproductive Medicine, 2011).

It is not certain yet whether the presence of oocytes with SERa may impact the clinical outcome of an ICSI cycle. More and more publications report similar embryological and clinical outcomes between SERa positive and SERa negative oocytes and cycles (Hattori et al., 2014; Itoi et al., 2017; Mateizel et al., 2013; Setti et al., 2016; Shaw- Jackson et al., 2016; Sá et al., 2011). In addition, recent publications show no significant difference in (major) malformation rate per live birth for SERa positive cycles compared to SERa negative cycles (Ebner et al., 2008; Hattori et al., 2014; Itoi et al., 2017; Mateizel et al., 2013; Shaw-Jackson et al., 2014; Sá et al., 2011).

In this study, prognostic factors, and clinical outcomes of ICSI cycles with a different SERa positive oocyte proportion were investigated. Since the literature is limited on the influence of the proportion of SERa positive oocytes on cycle outcomes when SERa positive oocytes are discarded and not used for ICSI.

## Methods

### Patients

This study retrospectively investigated ICSI cycles with ovum pick-up (OPU) between January 1, 2016, and December 31, 2019, that were performed at University Hospital Ghent, Belgium. Only complete in-house cycles were included, excluding oocyte donation and mixed ivf/ICSI cycles. The oocytes were evaluated for the presence of SER aggregates at the time of ICSI using an inverted microscope with Hoffman modulation contrast microscope. The SERa positive cycles have at least one SERa positive metaphase II (MII) oocyte and SERa negative cycles contain no affected MII oocytes. The data of the obtained cycles are divided into three groups based on the proportion of SERa positive oocytes to the total number of MII oocytes, namely none (0%), low (<30%) and high (≥30%) proportion of SERa positive oocytes.

### Ovarian stimulation and oocyte retrieval

The ovarian stimulation was done by menotrophin (Menopur ® ; Ferring, Hoofddorp, The Netherlands), corifollitropin alfa (Elonva® , MSD, Oss, the Netherlands), follitropin alfa (Bemfola® , Gedeon Richter, Budapest, Hungary), follitropin beta (Puregon ®; MSD, Oss, the Netherlands), follitropin delta (Rekovelle ®, Ferring, Copenhagen, Denmark) or clomiphene citrate (Clomid ® , Sanofi Belgium, Diegem, Belgium) or a combination of the above. Pituitary inhibition was achieved by GnRH agonist (short or long protocol) triptorelin; (Decapeptyl®; Ferring, Hoofddorp, The Netherlands or Gonapeptyl ®, Ferring, Hoofddorp, The Netherlands) or GnRH antagonist cetrorelix (Cetrotide®, Merck Serono, Geneva, Switzerland) or ganirelix (Orgalutran®, MSD, Oss, the Netherlands). There were no statistical analyses made within the variable total dose of gonadotrophin, for follitropin delta, corifollitropin and clomiphene citrate due to the low number of observations.

Ovulation was triggered using 5000 IU of chorionic gonadotropin (hCG) (Pregnyl®; MSD Oss, the Netherlands or Ovitrelle ®, Serono Benelux, London, UK) or 0.2 mg of triptorelin 0.2mg (Decapeptyl®; Ferring, Hoofddorp, The Netherlands or Gonapeptyl®, Ferring, Hoofddorp, The Netherlands) in case of risk of overstimulation. The ovulation trigger was scheduled when three or more oocytes reached a size of 17-18 mm. OPU was performed 35 hours after the ovulation trigger.

### Oocyte preparation and ICSI

The obtained oocytes were placed in fertilisation medium (Cook, Bloomington, IN, USA) for 2 hours. Afterwards, the cumulus cells were removed via ICSI Cumulase (Cooper Surgical, Måløv, Denmark). The oocytes were assessed using an inverted microscope with Hoffman modulation contrast microscope. Oocytes from which the first polar body had been extruded were considered mature (metaphase II oocytes) and could be used for ICSI. At this stage, oocytes were evaluated for the presence of SER aggregates and were excluded from injection for ICSI in case of SER aggregates.

### Embryo culture and selection

After ICSI, the injected oocytes were cultured in Cleavage media (Cook, Bloomington, IN, USA) in a 6% CO2, 5% O2 and 89% N2 incubator at 37°C (Binder 210, VWR). Fertilisation was assessed 16-19 hours after ICSI. From day 3, embryos were placed in Blastocyst medium (Cook, Bloomington, IN, USA) under the same conditions. Embryo morphology was checked daily until the day of transfer or cryopreservation.

On day 2 and 3, embryos were evaluated based on number of blastomeres, the rate of fragmentation and the presence of multinucleation. On day 4, embryo compaction was also assessed. Gardner and Schoolcraft classification was used for the assessment on day 5 (Hardarson et al., 2012).

Ultrasound guided embryo transfer was routinely performed on day 5 using a Cook embryo replacement catheter (Sydney IVF, Cook, USA).

### Cryopreserved embryo transfer cycles

The transfer of a cryopreserved embryo took place in a spontaneous or artificial cycle using oestradiol valerate (Progynova®, Bayer, Diegem, Belgium) and progesterone (Utrogestan®, Besins Healthcare, Brussels, Belgium).

### Outcome parameters

Three sets of variables were compared for these three groups, none (0%), low (<30%) and high (≥30%) proportion of SERa positive oocytes, namely the patient characteristics, cycle characteristics and clinical outcomes.

The clinical outcomes were considered cumulative per cycle. An embryo on day 5 was transferred or cryopreserved if it was at least an early blastocyst stage 1. The transfer cancellation is the percentage of cycles without transfer, in which oocytes were obtained, excluding PGT cycles. The fertilisation rate is the ratio between the number of zygotes and the number of oocytes injected. The blastocyst rate is the ratio between the total number of transferred and cryopreserved embryos and the number of zygotes. The implantation rate is the ratio between pregnancies with positive human chorionic gonadotropin (hCG) and the number of embryos transferred. The live birth rate is the number of children born expressed per number of embryos transferred.

### Data analysis and statistics

Different tests were performed for continuous variables and categorical variables for the predictive variables and cycle outcomes. The Kolmogorov-Smirnov normality test indicated that all continuous study variables deviate significantly from the normal distribution. Since several groups were compared, a Kruskall-Wallis test was performed per variable. In the event of a significant result, a Mann-Withney U test further examined differences between the statistical distribution of the two groups. The differences between groups for categorical parameters were analysed via a chi- square test. In addition, a multivariate analysis was performed with the variables that have significant differences. For the continuous variables, a multivariate regression analysis was performed. For the categorical variable “no transfer” a logistic regression analysis was performed. The analyses were performed using SPSS 27. A P value < 0.05 was considered to be statistically significant.

## Results

A total of 2468 in-house ICSI cycles with ovum pick-up (OPU) between January 1, 2016, and December 31, 2019, were included (Table I). The proportion of cycles with at least 1 SERa positive oocyte (SERa positive cycles) is 15 percent (n=371). The data is furthermore divided into three groups based on the proportion of SERa positive oocytes in relation to the total number of MII oocytes: no SERa positive oocytes (SERa negative cycles, n=2097), <30% SERa positive oocytes (SERa low proportion cycles, n=262), ≥30% SERa positive oocytes (SERa high proportion cycles, n=109).

**Table I:** Descriptive statistics of the included cycles.

		Mean	Range
Patient characteristics			
Maternal age (year)		34.5	19 – 44
BMI		24.1	16.01 – 40.17
AMH (ng/ml)		2.3	0.04 – 19.20
Cycle rank		2.9	1 – 21
Gravidity		0.9	0 – 13
Parity		0.3	0–6
Cycle characteristics			
Stimulation protocol			
	% urinary gonadotrophines	76.7 (n=1891)	
	% recombinant gonadotrophines	20.8 (n=514)	
Pituitary suppression protocol			
	% long agonist	4.3 (n=107)	
	% short agonist	63.9 (n=1575)	
	% antagonist	30.3 (n=746)	
Ovulation trigger			
	% human chorionic gonadotrophin	94.0 (n=2317)	
	% triptorelin	4.5 (n=112)	
Total dose of gonadotrophines			
	Menotropin (IE)	2863 (n=1890)	400-9225
	Follitropine alfa (IE)	2809 (n=260)	500-5475
	Follitropine bèta (IE)	2617 (n=134)	875-5100
Days of stimulation		13.5	6 - 34
Serum oestradiol at trigger (pg/ml)		2206	42.1 - 9240
Serum progesterone at trigger (pg/ml)		0.80	0.15 - 19
% SERa positive cycles		15 (n=371)	
Clinical outcome			
Oocytes per OPU		11.4	0 - 59
Injected oocytes		8.3	0 - 37
Good quality embryo at day 5		2.4	0 - 22
Transfer cancellation (%)		23.7	
Fertilization rate		0.69	0-1
Implantation rate		0.42	0-1
Live birth rate		0.26	0-2

### Patient characteristics

A significant difference was observed between the groups for maternal age and AMH. The women with a high SERa positive oocyte proportion were generally older (36.2 years for the SERa high proportion cycles vs. 34.5 years for the SERa negative cycles, p < 0.001) and had a lower AMH levels (1.6 ng/ml vs. 2.3 ng/ml, p < 0.001) relative to the women with SERa negative cycles. The SERa low proportion cycles showed opposite results: the women were younger (33.8 years for the SERa low proportion cycles vs 34.5 years for the SERa negative cycles, p = 0.04) and had higher AMH values (2.6 ng/ml vs 2.3 ng/ml, p < 0.001). For the other characteristics, namely BMI, number of attempts, gravidity, parity and indication, no significant difference was observed (Table II).

**Table II:** Patient characteristics of cycles with a different proportion of SERa positive oocytes; P value (P): between groups, 1 between no-low SERa, 2 between no-high SERa, 3 between low-high SERa. * = significant.

		SERa negative (n=2097)	SERa positive low (<30%) (n= 262)	SERa positive high (≥30%) (n= 109)	P
Maternal age (year)		34.5	33.8	36.2	<0.001* 0.041* <0.001^2^* <0.001^3^*
BMI		24.1	24.5	24.3	0.456
AMH (ng/ml)		2.3	2.6	1.6	<0.001* <0.001^1^* <0.001^2^* <0.001^3^*
Cycle rank		2.8	2.9	3.1	0.323
Gravidity		0.92	0.96	0.74	0.634
Parity		0.25	0.30	0.22	0.644
Indication					
	Maternal age	9.5%	8.4%	13.8%	0.266
	Uterus anomaly	2.7%	4.2%	2.8%	0.372
	Ovarian factor PCOS	5.0%	6.5%	0.9%	0.078
	Ovarian factor POI	1.2%	0.0%	0.9%	0.202
Recurrent miscarriage		5.8%	5.3%	5.5%	0.957
	Endometriosis	9.2%	6.1%	8.3%	0.254

### Cycle characteristics

A difference in the total dose of gonadotrophins was observed when menotrophin was used, namely a higher dose was administered to women with a SERa high proportion cycle (3227 IU for the SERa high proportion cycles vs 2858 IU for the SERa negative cycles, p < 0.001). The SERa low proportion cycles showed higher serum oestradiol levels at ovulation trigger (2602 pg/ml for the SERa low proportion cycle vs 2167 pg/ml for the SERa negative cycles, p < 0.001). The mean serum progesterone at trigger was higher in all SERa positive cycles vs. SERa negative cycles (0.78 pg/ml for SERa negative cycles vs. 0.91 pg/ml for SERa low proportion cycles vs. 0.87 pg/ml for SERa high proportion cycles, p = 0.001, p = 0.001). Regarding the pituitary suppression protocol, a significant difference was seen between the three groups for short agonist (63.7% for SERa negative cycles vs. 60.3% for SERa low proportion cycles vs. 77.1% for SERa high proportion cycles, p = 0.008) and antagonist (30.6% for SERa negative cycles vs. 32.1% for SERa low proportion cycles vs. 18.3% for SERa high proportion cycles, p = 0.020). No significant differences were observed in the variables total days of stimulation, drug protocol and ovulation trigger when using human chorionic gonadotropin (Table III).

**Table III:** Cycle characteristics of cycles with a different proportion of SERa positive oocytes; P value (P): between groups, 1 between no-low SERa, 2 between no-high SERa, 3 between low-high SERa. * = significant.

		SERa negative (n=2097)	SERa positive low (<30%) (n= 262)	SERa positive high (≥30%) (n= 109)	P
Drug protocol					
	% urinary gonadotrophines	77.2 (n=1618)	74.4 (n=195)	73.4 (n=80)	0.434
	% recombinant gonadotrophines	20.4 (n=247)	23.3 (n=61)	23.9 (n=26)	0.399
Suppression protocol					
	% short agonist	63.7 (n=1335)	60.3 (n=158)	77.1 (n=84)	0.008*
	% long agonist	4.2 (n=89)	5.0 (n=13)	4.6 (n=5)	0.858
	% antagonist	30.6 (n=642)	32.1 (n=84)	18.3 (n=20)	0.020*
Ovulation trigger					
	% human chorionic gonadotrophin	94.1 (n=1974)	91.2 (n=239)	97.2 (n=106)	0.059
	% triptorelin	4.3 (n=91)	7.3 (n=19)	1.8 (n=2)	0.039*
Total dose of gonadotrophins					
	menotrophin (IE)	2858 (n=1616)	2748 (n=193)	3227 (n=81)	0.002*
					0.308^1^
					<0.001^2^*
					<0.001^3^*
	Follitropin alfa (IE)	2825 (n=218)	2643 (n=25)	2856 (n=17)	0.789
	Follitropin bèta (IE)	2576 (n=113)	2741 (n=16)	3145 (n=5)	0.402
Total days of stimulation		13.5	13.5	13.6	0.946
Serum estradiol levels at ovulation trigger (pg/ml)		2167	2602	2006	<0.001*
					<0.001^1^*
					0.131^2^
					<0.001^3^*
Serum progesterone at trigger (pg/ml)		0.78	0.91	0.87	0.001*
					0.001^1^*
					0.038^2^*
					0.969^3^

### Univariate analysis of cycle outcomes

After ovarian hyperstimulation, SERa high proportion cycles had a lower number of oocytes at OPU (9.1 for the SERa high proportion cycle vs 11.1 for the SERa negative cycles p < 0.001), a lower number of injected oocytes (4.2 vs 8.2, p < 0.001), the cycle ended more often without any embryo transfer (47.7% vs. 23.7%, p < .0.001) and lower implantation rates (0.30 vs 0.43, p = 0.016) compared to SERa negative cycles (Table IV).

**Table IV:** Cycle outcomes of cycles with a different proportion of SERa positive oocytes; P value (P): between groups, 1 between no-low SERa, 2 between no-high SERa, 3 between low-high SERa. * = significant.

	SERa negative (n=2097)	SERa positive low (<30%) (n= 262)	SERa positive high (≥30%) (n= 109)	P
Oocytes per OPU	11.1	15.1	9.1	<0.001*
				<0.001^1^*
				<0.001^2^*
				<0.001^3^*
Injected oocytes	8.2	10.5	4.2	<0.001*
				<0.001^1^*
				<0.001^2^*
				<0.001^3^*
Good quality day 5 blastocysts	2.3	3.2	1.2	<0.001*
				<0.001^1^*
				<0.001^2^*
				<0.001^3^*
Transfer cancellation (%)	23.7 (n=496)	14.9 (n=39)	47.7 (n=52)	<0.001*
Fertilisation rate	0.68	0.71	0.69	0.562
Blastocyst rate	0.41	0.41	0.41	0.653
Implantation rate	0.43	0.39	0.30	0.030*
				0.0206^1^
				0.016^2^*
				0.112^3^
Live birth rate	0.27	0.23	0.26	0.346

### Multivariate analysis of cycle outcomes

A multivariate regression model adjusted for age, progesterone level at ovulation trigger and type of pituitary suppression showed that SERa high proportion cycles resulted in fewer injected oocytes compared to SERa negative cycles (p < 0.001), a lower number of blastocysts on day 5 (p < 0.001) and a higher probability of embryo transfer cancellation (p < 0.001). In contrast to the univariate model, the number of oocytes obtained, and implantation rate did not reach significant difference (p = 0.097, p = 0.105) in the multivariate model. Furthermore, there was no significant difference in neither fertilisation, blastocyst, nor in live birth rates.

The analysis between SERa negative cycles vs. SERa low proportion cycles, adjusted for age, progesterone level at ovulation trigger and type of pituitary suppression, showed results similar to those of the univariate analysis. There were more obtained oocytes (p < 0.001), more oocytes available for ICSI (p < 0.001), more good quality blastocysts on day 5 (p < 0.001) and there was a lower risk of ending the cycle without embryo transfer (p = 0.023). Again, there was no significant difference in the fertilisation, blastocyst, implantation, and live birth rates.

## Discussion

Despite the data published to date, the clinical impact of ICSI cycles with SERa positive oocytes and the use of these oocytes for ICSI remains controversial. Our study investigated the prognostic factors for obtaining SERa positive oocytes at pick-up and the effect of the proportion of SERa positive oocytes on the clinical outcomes of such an ICSI cycle.

The key findings of this research are that cycles with ≥30% SERa positive oocytes tend to be more prevalent in women undergoing ovarian stimulation at a higher maternal age and in patients with a lower ovarian response. Treatment cycles with ≥30% SERa positive oocytes are less likely to result in an embryo transfer. However, live birth rate per transfer is not affected by the proportion of SERa positive oocytes.

Cycles with a low proportion of SERa oocytes (<30%) are more prevalent in the prognostically more favourable patients. The women in this category are younger and have higher AMH levels. They have higher oestradiol levels at the time of the ovulation trigger, consistent with a higher number of oocytes obtained at OPU. The proportion of oocytes suitable for ICSI (MII oocytes without SER aggregates) is also higher, as well as a higher number of good embryos on day 5 and fewer cycles end without embryo transfer. It seems that in this category SERa positive oocytes arise as a by- product during ovarian hyperstimulation and that they have no negative impact.

In contrast, SERa high proportion cycles (≥30%) occur in patients with a generally poorer prognosis. A higher average maternal age and lower AMH levels are seen in this group. They have received a higher dose of gonadotrophins (menotrophin), which is consistent with their average lower ovarian reserve. They show lower oestradiol levels at the time of ovulation trigger. In these cycles, fewer oocytes are injected, fewer embryos are suitable for embryo transfer or cryopreservation on day 5, and there are more cycles without embryo transfer.

The results regarding pituitary suppression are probably related to patient characteristics in the different categories. Historically, a short agonist schedule was more often used in older women with a lower AMH, as in the group with a high proportion of SERa positive oocytes. This is also seen in our dataset. In the short agonist group, the maternal age was significant higher and the AMH level was significant lower compared to the antagonist group (35.35 years for short agonist vs. 32.56 years for antagonist, p < 0.001 and 1.59 ng/ml vs. 3.79 ng/ml, p < 0.001). In a logistic regression analysis, the type of pituitary suppression is not independently related to the proportion of SERa positive oocytes. No relevant conclusions can be made about ovulation trigger triptorelin and dose of gonadotrophins follitropin beta due to the low number of observations in these groups.

Our results for implantation and live birth rates are consistent with the earlier data (Hattori et al., 2014; Mateizel et al., 2013; Sá et al., 2011). For prognostic factors, embryo quality and fertilisation, our data are difficult to compare with other studies since in this study a distinction is made according to the proportion of SERa positive oocytes. To our knowledge, this has only been investigated by Gurunath et al. (2019). They found a similar fertilisation rate and a lower number of good quality embryos available for transfer as the proportion of SERa positive oocytes increases, like we observed. Contrary to our findings, they reported a gradual reduction in live birth rate when the proportion of SERa positive oocytes per cycle is higher. However, the differences in the study by Gurunath et al. (2019), are not significant and are based on very low numbers.

Several reports suggest that inadequate ovarian stimulation is related, and possibly even causal, to the occurrence of SERa positive oocytes. In particular, for stimulations of longer duration, with a higher dose of gonadotrophins and more follicles and oocytes a higher risk of SERa positive oocytes is seen (Ebner et al., 2008; Gurunath et al., 2019; Hattori et al., 2014; Restelli et al., 2015; Setti et al., 2016; Sá et al., 2011). Data in this study shows a relationship between SERa low proportion cycles and ovarian stimulation. A low proportion of SERa positive oocytes occurs more often in high responders with a pronounced hyperstimulation, with higher oestradiol values at the time of ovulation trigger and a higher number of oocytes retrieved. SERa high proportion cycles occur more often in the patients with poor ovarian response, where higher doses of gonadotropins are administered, and fewer oocytes are retrieved from OPU. The link between higher doses of gonadotropins and the occurrence of SERa was described earlier (Ebner et al., 2008; Hattori et al., 2014).

Based on our results, once embryo transfer is performed, live birth rates are similar between the SERa negative and SERa positive cycles, regardless of the proportion of SER aggregates. The presence of SER aggregates in the cohort of oocytes in these cycles does not seem to influence the potential of the unaffected oocytes and embryos. The cycles with a high proportion of SERa positive oocytes result in a lower embryo utilization rate on day 5 and more transfer cancellations.

The fetal and neonatal consequences of SERa positive oocytes are still under debate. It remains unclear whether there is an association between SER aggregates and congenital malformations.

First, because the reported congenital abnormalities resulting from a transfer of a SERa positive cycles are so diverse. Second, these congenital abnormalities were frequently the result of a transfer of a non-affected embryo (originating from a non-affected oocyte) within a SERa positive cycle (Ebner et al., 2008; Otsuki et al., 2004; Sfontouris et al., 2018). Third, Ebner et al. (2008), Hattori et al. (2014), Itoi et al. (2017), Mateizel et al. (2013) and Sá et al. (2011) found no significant difference in (major) malformation rate per live birth for SERa positive cycles compared to SERa negative cycles.

Cycles with a high proportion SERa positive oocytes end more often without an embryo transfer (47.7%), because no usable embryos are present on day 5, partly due to the local policy to discard SERa positive oocytes. In addition, the recurrence risk in literature of having SERa positive oocytes in a subsequent cycle is estimated to be as high as 40% (Hattori et al., 2014; Itoi et al., 2017; Mateizel et al., 2013). For a patient, a cycle in which no transfer is possible is experienced as very negative and demotivating to continue the treatment. The latter leads to premature and unjustified treatment dropout (Verberg et al., 2008).

Given all the above arguments, the inclusion of SERa positive oocytes for ICSI should be further investigated.

In conclusion, the cycles with a high proportion of SERa positive oocytes face more often transfer cancellation. However, clinical outcomes per embryo transfer are not affected by the presence and proportion of SERa positive oocytes. The presence of SER aggregates in the cohort of oocytes in SERa positive cycles does not seem to influence the potential of the unaffected oocytes and embryos.
